# Patient satisfaction in inpatient psychiatric treatment compared with inpatient equivalent home treatment in Germany: an in-depth qualitative study

**DOI:** 10.3389/frhs.2023.1195614

**Published:** 2023-06-29

**Authors:** Nele Adam, Melanie Neumann, Friedrich Edelhäuser

**Affiliations:** ^1^ Faculty of Health, Department of Psychology/Psychotherapy, University of Witten/Herdecke, Witten, Germany; ^2^ Faculty of Health, Department of Medicine, Institute of Integrative Medicine and Integrated Curriculum for Anthroposophic Medicine (ICURAM), University of Witten/Herdecke, Witten, Germany; ^3^ Faculty of Health, Department of Medicine, Chair on Psychosomatic Medicine and Psychotherapy, University of Witten/Herdecke, Witten, Germany; ^4^ Gemeinschaftskrankenhaus, Department of Early Rehabilitation, Herdecke, Germany

**Keywords:** home treatment, psychiatric inpatient treatment, psychiatric inpatient equivalent home treatment, patient satisfaction, qualitative study

## Abstract

**Background:**

Inpatient treatment (IT) is the predominant form of psychiatric care in Germany and worldwide, whereby forms of psychiatric treatment have mainly evolved in the direction of home services. Inpatient equivalent home treatment (IEHT) is a new and additional pillar of psychiatric acute care provision legally embedded since 2018 in Germany.

**Objective:**

The aim of this study was to conduct an in-depth exploration as little qualitative research has been performed so far in Germany to examine possible differences in patient satisfaction with IT compared with IEHT.

**Methods:**

In the current qualitative study, *N* = 9 patients of a German hospital providing IT and IEHT were interviewed with the problem-centered interview. Inclusion criteria were IT or IT with subsequent IEHT. The theoretical sampling method was applied to select test persons in the research process. The experiences of the participants during their psychiatric treatment were analyzed using a qualitative content analysis.

**Results:**

The results of both types of psychiatric treatment refer to different satisfaction factors during the treatment period. The function of fellow patients, the setting of the treatment, the conditions in place, and the relationship to relatives turn out to be pivotal for patient satisfaction. In addition, the quality of the therapy and relationship to caregivers itself can have an impact on patient satisfaction, particularly by shared decision making. During the IEHT, patient satisfaction can be strengthened by the possibility to handle daily tasks, to be close to relatives, while not so close to fellow patients, whereas IT patients are mostly satisfied because of the distance to their everyday life and the closeness to fellow patients. The choice of the form of psychiatric treatment according to the individual needs of the patients seems to be one key driver that can in turn increase patient satisfaction. In addition, a clean and hygienic environment seems to be critical for our respondents as a lack of it is one of the reasons to drop out of treatment.

**Conclusions:**

Despite its limitations, this hypothesis-generating study is one of the first investigating German IEHT in comparison with IT in an in-depth qualitative approach contributing to a patient-oriented and cost-effective psychiatric treatment. Although hospitals are highly complex organizations and therefore not directly comparable, other German and international providers of IEHT may derive several generic success factors from this study for the development and improvement of patient satisfaction.

## 1. Introduction

Inpatient treatment (IT) is still the predominant form of psychiatric treatment, but in the last decades, it has mainly evolved in the direction of home treatment (HT) (1–5). According to the recommendation of international and national guidelines (6–8), many high-income countries have successfully implemented outreach mental health services for acute psychiatric care. In 2018, a new German law enabled national mental healthcare providers to implement team-based crisis intervention services on a regular basis, allowing for different forms of inpatient equivalent home treatment (IEHT) (9–11). The German IEHT is in parts related to the internationally known home treatment or crisis resolution teams (CRT). It provides acute psychiatric treatment with a similar intensity and flexibility to IT, but delivered in the home of the users by mobile, multi-professional, and permanently available teams (e.g., from nursing care, ergotherapy, psychotherapy) (12). The key elements are daily home visits, medical rounds by mental health specialists, regular multi-professional team meetings, and a round-the-clock availability of the team or the hospital (13). The patients in Germany receive IEHT either as alternative or following an IT (10). Moreover, in adherence to the systemic therapeutic approach (14), close relatives are integrated as well into the IEHT (15).

Due to the short time period since the legal implementation of IEHT in Germany, there is a lack of detailed evaluation. Although there exist several studies on patient views (16, 17), as well as systematic reviews (18) on HT and CRT from other countries, their evidence is for limited use as IEHT is a unique construct within the German healthcare system and fulfills only some of the internationally defined criteria for HT or CRT (19). It differs, for example, from HT in Great Britain as it is less flexible and requires at least one personal contact with the users per day, cannot be gradually phased out, and is associated with strict criteria for reimbursement. Moreover, it cannot be compared with the assertive community treatment as these services have been constructed for long-term support, in contrast to the limited scope of IEHT, which is restricted for acute crisis (18–20).

To the best of our knowledge, there exist currently only two published German studies on this topic based on one data set. The first published study is the one of von Peter and colleagues (21) exploring IEHT on patients, informal caregivers, and staff in 12 psychiatric hospital departments. In their mixed-method process evaluation, the authors found that the evaluations of the patients were largely influenced by the advent of continuous forms of care, better accessibility, and by their degree of autonomy in steering of their services. Specifically, their qualitative analysis (21) did show that the patients valued IEHT for its potential to deal with “embedded and real-life problems,” instead of receiving treatment “in a greenhouse” (on a ward), perceived to be “normalizing and de-stigmatizing” although at the risk of having the potential to “disrupt a person's or family's privacy” (21, p. 9). Moreover, continuity of care was highly valued and being experienced as leading to “more trustful relationships” between staff, patients, and their families as well as to a “more solid and nuanced understanding” between them. Also, autonomous steering of services and flexible care management were both perceived to lead to more “need-adapted forms of treatments.” Being able to “choose ones’ own treatment setting” was perceived to increase “personal empowerment” (21, p. 9). The same research group published more details of the qualitative part of their study with *N* = 13 patients in a German journal (22): IEHT seems to strengthen the integration of the everyday life of the patients and the treatment flexibility as it is more focused on the individual needs of the patients. Also, the quality of therapy was perceived, e.g., more on “eye level” with the therapist, and therapy time was sensed more intensively. After having experienced IEHT once, the patients generally rated it better. The disadvantages named by the patients were, e.g., the lack of daily structure, lack of contact with fellow patients, and being alone with themselves. Moreover, both family members and patients experienced the therapists being in their homes, in some cases, as “crossing a border” (22, p. 4–5).

The study protocol by Baumgart and colleagues (19) describes a similar research aim: a naturalistic, quasi-experimental cohort study to evaluate IEHT in 10 German hospitals within a multi-method research approach to evaluate the experiences of the stakeholders of care, service use, efficacy, costs, treatment processes, and implementation processes of IEHT from different perspectives. However, the results of this study are not published yet. A further interesting study protocol is the one of Reinke and colleagues (23). In their randomized controlled trial, IEHT with peer support (i.e., the people with lived experience of a mental illness are trained to support others on their way toward recovery) is compared with IEHT without peer support within a network of eight psychiatric clinical centers in Germany. The authors (23) give also an overview of studies on peer support in psychiatric care with promising results for patient-reported outcomes (PROs).

An older German quantitative study on a model project—before the new German IEHT law was introduced in 2018—comparing patient satisfaction in adolescents with mental illness and their parents in IT and IEHT concludes that adolescents are more satisfied with the IT compared with patients with IEHT. However, the adolescents with treatment experience of both IEHT and IT report significantly higher satisfaction with IEHT compared with preceding IT (24), similar as in (22).

Nevertheless, as more and more psychiatric departments in Germany and worldwide are expected to include forms of IEHT into their treatment options, evidence on its effectiveness, i.e., also on PROs (25), are of high relevance (18) for their acceptance. Batbaatar and colleagues (26, 27) conclude in their systematic reviews that patient satisfaction is a crucial and multidimensional PRO and a widely measured indicator in the evaluation of healthcare service quality as the patients have contributor, target, and reformer roles in quality assurance. The results of patient satisfaction surveys allow the healthcare providers to identify problems and service factors that need improvement. It also enables the policymakers to understand the needs of the patients and, consequently, to make strategic plan for effective and better-quality services, e.g., improves service management and behavior of health professionals. Moreover, a high level of satisfaction results in the decision of the patients to choose a health service and to have an intention to return to a particular hospital (26).

Furthermore, a higher patient satisfaction with healthcare services changes the behavioral intentions of the patients, such as compliance with the doctor's recommended treatment and appointments to follow-up, which results in better health outcomes (27). Satisfaction may be strengthened through participation opportunities in the decision-making process that match individual preferences (28). However, the strongest determinants of patient satisfaction across studies in the review of Batbaatar and colleagues (27) were the quality of the interpersonal skills and competence of the healthcare providers and the physical environment of the facility, accessibility, continuity of care, hospital characteristics, and outcome. Among them, the interpersonal care quality of the health providers was the essential determinant of patient satisfaction.

In summary, patient satisfaction is seen as an important criterion in the evaluation of (psychiatric) healthcare (29) since an appreciative relationship between patients and caregivers is not only associated with patient satisfaction (24) but seems to be vital for successful treatment results and for utilization of the services (once again).

## 2. Aim of the study

The forms of IEHT are of high importance in an innovative and cost-effective psychiatric healthcare, and patient satisfaction plays on several levels a significant role for the quality of care and thus for patients' acceptance and utilization of (forms of) IEHT. Since the introduction of the new German IEHT law in 2018, in-depth comparisons of IEHT in contrast to IT are rare in Germany: Although there exists the mixed-method process evaluation of von Peter et al. (21, 22) containing a qualitative part, but they are not comparing IT vs. IEHT and not providing a comprehensive report on their qualitative results. Therefore, the aim of the present study was to close this research gap and conducting in-depth qualitative research to examine possible differences in patient satisfaction from the subjective perspective of two groups of German patients experiencing IT and IEHT.

## 3. Methods

### 3.1. Study design

This qualitative study was designed to gain a profound understanding and overview of interpersonal differences in intrapersonal perceptions, experiences, and feelings in connection with the research aim mentioned above. Moreover, a qualitative study is particularly appropriate given that the research topic has been scarcely researched. We chose a descriptive phenomenological research perspective (compare a detailed overview in 30): for an appropriate consideration of subjective meanings, individual interpretations, and the context of actions and opinions as described by the participants. That means specifically, we “describe the phenomena phenomenologically, rather than explaining them” (30). In accordance with this approach, it was decided to conduct semi-structured interviews based on Witzel and Reiter (31) and to apply an inductive qualitative content analysis according to Mayring (32).

The authors of this paper are following the Standards for Reporting Qualitative Research (SRQR) guidelines (33).

### 3.2. Interview guideline and data collection

The approval for this qualitative study was obtained from the ethics committee of Witten/Herdecke University (Germany) prior to the interviews (application no. 43/2019). In addition, the test persons signed informed consent declarations to participate in the research project. Individual code numbers were assigned to each test person as requested by the ethics committee.

The interview guide was drawn up according to Helfferich (34) with the aim to register individual differences in patient satisfaction with the different treatment forms of IT and IEHT as accurately as possible. The focus in the interview for both groups was, e.g., on positive and negative experiences with therapy, caregivers, fellow patients, and family; also on expectations, recommendations of the form of treatment, suggestions for improvement, etc. (for complete interview guideline, see Supplementary Material).

The interviews were conducted based on the “problem-centered interview” that according to Witzel and Reiter (31) takes place, e.g., in the own home of the patients or a room at Witten/Herdecke University (see Table 1). The aim of the guideline-based problem-centered interview is an unbiased recording of the individual actions as well as subjective perceptions and processing modes. Its main features are the following: (1) narrative-generating communication strategies including conversation starters, guiding questions, and *ad hoc* questions and (2) comprehension-generating communication strategies allowing, e.g., for reflections back to the statements of the interviewees, comprehension questions, and confrontations in case of contradictions and/or evasive answers.

**Table 1:** Sample description.

** **	**Inpatient treatment (IT)**	**Inpatient equivalent home treatment (IEHT)**
Female	4	5
Male	0	0
Range of age	20–30	30–65
Duration of therapy	Between 3 and 51 days	Between 11 and 38 days
Average interview duration	55 min	70.8 min
Diagnoses	Obsessive-compulsive disorder, recurrent depression, combined personality disorder, bulimia	Schizoaffective disorder, psychosis, depression, anxiety and panic disorder, complex PTSD, eating disorder, ADHS

PTSD, post-traumatic stress disorder; ADHS, attention deficit/hyperactivity syndrome.

From 13 August to 26 November 2019, the interviews were conducted and recorded on audio equipment. The study persons were offered subsequent support by their therapists in the IT/IEHT in case of distress caused by the interview.

### 3.3. Recruitment of test persons

As a part of our inclusion criteria, we recruited people who had utilized IT or IT with subsequent IEHT at least once, were of the age of legal majority, able to speak and read the German language, could furnish written consent to participate, and had a physical and mental condition permitting conversation. The theoretical sampling method (35) was applied to select test persons in the research process. This means that first interviews provided indications of more or less satisfied patients who were then successively included in the study. This heterogeneity of patient satisfaction was meant to ensure increased information value. However, during recruitment, only the female respondents expressed interest to participate in the research project, and therefore, the sample was kept homogenous regarding gender throughout. The test persons were recruited via a notice board in the hospital and also via caregivers, who were informed prior by NA about the study, its aims, and the inclusion criteria.

The aim was to recruit *N* = 14–16 test persons for this qualitative study, but despite great efforts, only *N* = 9 female individuals indicated their interest in the research project (see Table 1). As a result, data saturation could not be ensured as we could only recruit female patients, thus missing data from a male perspective.

### 3.4. Sample description

The sample examined consists of nine women aged between 20 and 61 years, five of whom report experience with IEHT (and prior IT experience) and four with IT (without IEHT experience) of approximately the same treatment duration (see Table 1). No further sociodemographic data are reported here to preserve the anonymity of the participants.

### 3.5. Qualitative analysis

The interviews were fully transcribed using the transcription scheme of Dresing and Pehl (36). Data analysis was done with the German transcripts, and only the selected citations for the results section were translated into English, whereby the first authors did doublecheck its accuracy.

An inductive data analysis was performed using the qualitative content analysis method according to Mayring (32). That means, that the first author (NA) began by identifying text passages relating to the topic that were subsequently summarized and paraphrased to reduce data material to a practicable proportion without altering the main content. The main categories and subcategories were created successively and filled with quotations from transcribed interviews. The category system drawn up in this way was then applied to the entire data set to ensure a coherence of results.

The other first author (MN) conducted the same analyzing steps, and the last author (FE) independently reviewed the analyses of the first authors and conclusions. This involved careful review of the transcripts, themes, and results to ensure accurate formulation of conclusions from the individual interview data (investigators triangulation) (37). All disagreements over conclusions were intensively discussed in a group setting and adequately resolved. All authors were trained and experienced in qualitative data analysis as well as conducting and publishing qualitative research.

### 3.6. Personal reflexivity

A research based on a qualitative methodology recognizes the importance of locating and highlighting the central opinions informing the research process according to the Standards for Reporting Qualitative Research (33) and the APA Style JARS (38). Moreover, as we chose a descriptive phenomenological research perspective, reflexivity of the authors is especially of high importance.

This paper has been heavily informed by the standpoint of the first authors (NE and MN). The role of the last author (FE) was rather to assist the other authors throughout the research process and to also reflect and critically engage in their relationship and motivation for conducting the study and assess the ways in which it has informed the study aims, design, and analysis.

The two first authors (NA and MN) are both clinical psychologists and experienced qualitative researchers at the University of Witten/Herdecke, Germany. MN is also adequately skilled in qualitative evaluations and quantitative research and has practical knowledge in the German healthcare system due to her work as a nursing assistance for several years. Before the research project started, NA worked for a few months at the investigated ward/hospital, and MN had no prior connection to the research field. The last author (FE) is a neurologist and a qualitative and quantitative researcher with practical knowledge in the German healthcare system due to his work as a physician for nearly 30 years. He had no prior connection to the research field.

## 4. Results

**Figure 1:**
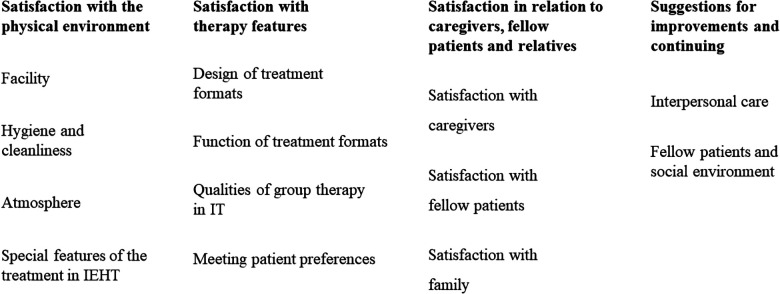
Overview of main categories and their subcategories.

The following sections describe the main and subcategories, and Figure 1 provides a graphical overview of the results.

### 4.1. Satisfaction with the physical environment

Design factors of the physical environment constitute an important element of patient satisfaction when analyzing data obtained from the respondents.

#### 4.1.1. Facility

A warm, welcoming and comfortable design of the IT setting seems to be important to test persons:

… well, because this is […] a hospital where you go. And that […] it should […] convey a sense of warmth despite the circumstances. Some sense of welcome (I5 receiving IT and IEHT, p. 10 l237–240).

The test persons criticized that the ward design should more be adapted to their need, e.g., window grids in particular are seen as “unhomely” and evoke feelings of being imprisoned. In contrast, the test persons in IEHT are in charge of designing their own home.

#### 4.1.2. Hygiene and cleanliness

A clean and hygienic environment contributes as well to the sense of well-being of the test persons, and a lack of it is one of the reasons to drop out of treatment. On the other hand, an exaggerated cleanliness can impair the sense of normality.

Regular visits from the caregivers in the context of IEHT induce the test persons to pay more attention to cleanliness and order at home and to “arrange things nicely for themselves as well” (I9 receiving IT and IEHT, p. 10 l249), so that “you feel more at ease” (I5 receiving IT and IEHT, p. 10 l249).

#### 4.1.3. Atmosphere

The atmosphere in IT is positively impacted by cleanliness, hygiene, a freshly renovated ward, and a relaxed climate:

But the people there and the social encounters made it better. The outward appearance of the clinic […], they have these cozy sitting areas, quite comfortable […] really help to create a relaxed atmosphere (I6 receiving IT, p. 5, l. 120–123).

Informal gatherings for shared meals are mentioned specifically as valuable and a quiet ambiance is much appreciated on these occasions.

On the other hand, own pressure of expectation of a test subject can lead to a tense atmosphere at home (in IEHT):

… this additional pressure, so that I think: Okay, this is something I want. So, I must […] become actively engaged. This means “creating framework conditions” so that my life and home circumstances will have the lowest possible negative impact on the therapy situation […] or the therapy conversation. This has put me under a lot of pressure (I7 receiving IT and IEHT, p. 17–18, ll. 442–446).

On the IEHT ward^‡^ the breakfast atmosphere depends on the fellow patients present and on their mood. The respondents also mentioned the colors and smells on that ward helping to create a warm and welcoming impression.

#### 4.1.4. Special features of the treatment in IEHT

“There is no place like home” (I3 receiving IT and IEHT, p. 4, l. 76): This is where the test persons generally feel good, “more familiar” (I4 receiving IT and IEHT, p. 3, l. 70), not cut off from their usual surroundings, and freer. Or they plan to create their own home, where there are fewer places with negative associations:

… when I was really ill, I was on the intensive ward and was not permitted to leave […], but whenever I saw that door [entrance to the intensive ward] then I […] always took distance. […] Now it is better (I3 receiving IT and IEHT, p. 21, ll. 515–519).

At the same time, there may be worries to create negative associations in one's own home:

…. if now for example there were a trigger somewhere to remind me of my couch at home, then I will later have a problem with my couch (I7 receiving IT and IEHT, p. 15, ll. 372–374).

It may also become challenging to meet the demands of several roles simultaneously in IEHT, such as the roles of the patient and of the mother. Building up a positive relation to the caregivers can help to reduce the stress involved. Nevertheless, the prospect of being accepted into the IEHT program in the near future has a relieving effect on the test subjects.

### 4.2. Satisfaction with therapy features

This category is frequently connected with the question as to which activities are conducted in which manner and to which purpose in the course of treatment. Therefore, this main category constitutes an important basis for the analysis of patient satisfaction.

#### 4.2.1. Design of treatment formats

In both formats of treatment, invigorating experiences play an important role in the healing process, such as learning new strategies and methods:

… this is […] valuable, when you see progress and can feel it (I5 receiving IT and IEHT, p. 25, l. 602), I have actually had positive experiences throughout (I5 receiving IT and IEHT, p. 29, l. 709).

Both forms of interventions aim to improve the quality of life, as one possible way toward normality. A clearly formulated and transparent therapy plan helps to achieve this objective. Other supportive elements can be an overview about which therapy targets the individual patient can reach over which treatment period, and a jointly planned schedule for a daily structure. Such a plan offers the test subjects a valuable structure:

… there also was a very clear professional distance with the clear understanding: Look, these […] are the points we discussed, right from the start, in the preliminary talks, this is what we aim to achieve […]… care was always taken to ensure that (I9 receiving IT and IEHT, p. 6, ll. 144–148).

All the test persons who defined treatment targets in consultation with their caregivers continued the treatment. Self-determined treatment objectives may therefore be assumed to be important factors for successful treatment. Another significant conclusion from the data analysis is that the expectation of positive results of the test persons is frequently associated with the actual treatment results.

#### 4.2.2. Function of treatment formats

The function of IT is to consciously provide a protected space away from the home setting and the opportunity:

… just to take a step back from daily life and the obligations involved […], […] that you listen to yourself and do something for yourself (I8 receiving IT, p. 12, ll. 284–286).

Where these functions are not fulfilled, the test persons may be inclined to break off the treatment prematurely and be discouraged from resuming treatment at a later time. In contrast, IEHT is rather intended to:

… make it easier to return home […], not to fall back into old patterns of behavior (I5 receiving IT and IEHT, p. 1, ll. 12,13).

The test persons describe IEHT as a highly individual form of intervention that can be adapted to their personal situation and circumstances. The focus is on the individual to be treated, and IEHT is therefore perceived as an intensive form of therapy:

… in IEHT you feel positively challenged to work on yourself all the time. You […] need not concern yourself with others because there is nobody else, I mean no other patients. […] You are very much faced with yourself (I9 receiving IT and IEHT, p. 25 l618–621).

IEHT provides a kind of support that is relevant to everyday life. However, coping simultaneously with daily life and treatment may present a particular challenge. It is therefore essential to keep an eye on the individual degree of challenge of the patients and achieve a positive completion of therapy:

… the time I was in therapy was more than enough and shouldn’t have lasted longer. I think the effect would have been reversed. […] It is too open and too intensive, and then I was really glad to be […] well within the time limit […] because […] it felt like a wound you reopen every day (I7 receiving IT and IEHT, p. 46, ll. 1171–1182).

#### 4.2.3. Qualities of group therapy in IT

Group therapies form part of IT (but not of IEHT) and are perceived as central elements in this treatment format. It enables the test subjects to express their emotions in different ways and switch their thoughts to different things. At the same time, the physical closeness to fellow patients in this setting encourages the impression of being understood, of being not alone:

There are others who feel the same way, although you would not think so judging them by appearance, people who understand you and, well, who can […] give helpful tips (I8 receiving IT, p. 11, ll. 268–270).

On the other hand, closeness to fellow patients may also cause difficulties in one's own therapy process:

In group therapies you get an idea of other people's innermost thoughts, and sometimes it is hard to keep your distance and […] remain true to yourself (I8 receiving IT, p. 6, ll. 147–149).

Cancellation of group therapy sessions by the hospital organization is, however, one of the most frequent points of criticism.

#### 4.2.4. Meeting patient preferences

In the context of therapy, a pattern among the test subjects becomes apparent with regard to patient satisfaction: the patients in IT are particularly satisfied when they hope for some degree of freedom from responsibility, when they leave their daily lives behind including the obligations involved, and when they develop a wish to be close to like-minded persons and fellow patients. In contrast, the test persons in IEHT are most satisfied with the option to recover at home in their familiar domestic surroundings and to receive psychiatric care while meeting responsibilities for close persons.

### 4.3. Satisfaction in relation to caregivers, fellow patients, and family

A third key category in the analysis of differences in patient satisfaction is the relationship with all persons connected to the therapy.

#### 4.3.1. Satisfaction with caregivers

In both treatment formats, the time and effort invested by caregivers and the patients' sense of being appreciated as a human being on a personal level have a favorable effect on patient satisfaction:

… obviously this […] can only work in a functioning relationship (I7 receiving IT and IEHT, p. 39–40, ll. 1008–1010).

Professionality is mentioned as a specific factor in this context—on the personal as well as the therapeutic level. Both levels are required for good cooperation. IT therapy should offer patients the chance to become actively involved in obtaining their treatment goals, in collaboration with their caregivers. A lack of opportunity for active participation generates discontent in the test subjects and may even induce them to early terminate the treatment. A sense of being abandoned despite the presence of caregivers—for example, due to shortage of time—can also enhance the feelings of dissatisfaction.

IEHT in contrast gives the test subjects the impression of being key agents:

… this is an entirely different approach […], where the affected person takes the lead, or becomes something like the ordering party […] where affected individuals […] are seen as experts on their own behalf (I7 receiving IT and IEHT, p. 42, ll. 1069–1073).

However, the staff usually rotates in IEHT, so that the patients need to build up relationships to unfamiliar caregivers, and this takes time. This may create a feeling in the patients that they cannot work on their objectives with a maximum of efficiency, which reduces the perceived therapeutic success and degree of patient satisfaction.

Evident dissatisfaction therefore requires that potential problems are openly addressed in both types of treatment in order to keep the patients satisfied and ultimately to ensure successful treatment results.

#### 4.3.2. Satisfaction with fellow patients

Physical proximity to fellow patients is perceived as essential specifically in IT, as their support may enhance patient satisfaction. “True friendships” (I1 receiving IT, p. 5, l. 124)—like in a “surrogate family” (I1 receiving IT, p. 8, l. 184)—may be retained after the hospital stay. The shared experience of a group during the therapeutic process may be a key factor for successful treatment. At the same time, close proximity to fellow patients may have adverse effects on patients:

… well in some respects it was good to have these contacts to fellow patients, but less so in others because […] certain fellow patients tended to trigger particular behaviors such as self-injury (I6 receiving IT, p. 11, ll. 251–254).

Strong feelings of compassion for others in IT therapy can create additional distress and distract the attention of the patients from tackling their own problems. They may also evoke detrimental thoughts about their personal situation. On the other hand, IT creates physical distance and offers the opportunity to enjoy peace and quiet, which again enhances patient satisfaction. In contrast, contacts with fellow patients in the context of IEHT—in the investigated IEHT ward only on Fridays during a breakfast—are described as comparatively reserved:

I met fellow patients only once, at that shared breakfast (…). I personally do not need that (I7 receiving IT and IEHT, p. 20, ll. 494, 495).

The greater distance in IEHT results in less compassion with fellow patients. This is one reason why the test subjects in IEHT wish for more contacts with others in the same situation.

#### 4.3.3. Satisfaction with family

IT perceived as challenging can serve to strengthen relations between the test persons and their family members and be used as an occasion to frankly discuss the disorder in question. Frequently, IT is intended to relieve the family:

… I would not wish to burden my family with this responsibility (I2 receiving IT, p. 9, ll. 224, 225).

In contrast, the patients in IEHT often feel the urge to meet obligations to immediate family or dependents even in stages of illness:

… therefore, it is extremely valuable for a mother […] to be there for the family and get the help you need yourself (I5 receiving IT and IEHT, p. 28, ll. 693–694).

### 4.4. Suggestions for improvement and continuing

We found useful suggestions for improvement and continuing on different levels in IT and IEHT, which may contribute to higher patient satisfaction.

#### 4.4.1. Interpersonal care

The IT patients derive specific satisfaction from the proximity and support of fellow patients, and regular offers of group and individual therapies enhance patient satisfaction in general:

There are people who think the same way, […] who can understand that and […] give tips (I8 receiving IT, p. 11, ll. 267–269)

Thus, there is a wish for sufficient personnel to ensure the provision of such interventions.

In the context of IEHT, the test subjects report specific satisfaction with regular support from their caregivers. They appear to feel most content with caregivers who are familiar with their individual problems, which permits efficient cooperation in the form of personalized assistance and advice. Assigning a permanent and not rotating team to each individual patient might help to reinforce this positive effect:

… when I really work with one person who […] knows what the problem is around me, that they could help me much more concretely […], then I realized that it would be good if […] two employees would always take care of one patient and not all employees […] of all patients. always take care of one patient and not all employees […] take care of all patients […]. (I9 receiving IT & IEHT, p.18, ll. 431–437)

Particularly gratifying elements, so the test subjects in both types of therapy, are friendly treatment by caregivers, a sense of being taken seriously, and the chance to become actively involved in the therapy process:

… where you are more the tone setter, or the contracting authority (I7 receiving IT and IEHT, p. 42, ll. 1070–1071)

A therapy plan providing a firm structure in daily life and a jointly drawn-up time schedule to reach individual therapy objectives offer valuable orientation and also contribute to patient satisfaction.

#### 4.4.2. Fellow patients and social environment

Some IEHT respondents wish for more contact with fellow patients and would welcome a wider range of collective activities, for example, after the shared breakfast on Fridays.

If you could perhaps offer one or two […] talk groups per week, where you could also go if you wanted to. So, on a voluntary basis, if all patients […] could exchange information. Like a self-help group perhaps within IEHT. (I9 receiving IT and IEHT, p. 29, ll. 720–723)

Continued adherence to the daily time schedule in IEHT would also contribute to patient satisfaction and successful therapy outcome because it helps the patients to be better prepared for visits from caregivers. Other ideas to improve IEHT refer to the following:

• The option of a meeting with the persons primarily involved in the therapy after the first half of the treatment period for a joint reflection on relations
• Increased networking with social services such as domestic help or “meals on wheels”
• Employment of more caregivers who have undergone psychiatric treatment themselves (peer support).

Another suggestion is an arrangement with IEHT patients prior to the start of the therapy as to which persons from their domestic environment (e.g., neighbors, acquaintances) should be allowed to know that the visiting caregivers are providing psychiatric care. Here, the idea is to avoid unintentional stigmatization.

## 5. Discussion

This hypothesis-generating qualitative study on patient satisfaction reveals three crucial differences comparing IT with IEHT. These concern, firstly, the different environments, secondly, the role of fellow patients, and thirdly, the role of family members. IT offers a protected environment away from daily life and the obligations involved, whereas the test subjects in IEHT feel freer and more familiar in their own domestic surroundings. In IT, relations to fellow patients are characterized by special proximity and have a significant supportive effect (due to regular group therapy sessions), whereas the patients in IEHT report a more reserved relationship and less compassion to fellow patients (due to breakfast meetings only on Fridays at the investigated facility) as a result, which can also mean a lower degree of distress and distraction. IEHT is probably perceived as very intensive and individual because contacts with fellow patients are limited. The family members of the patients in IT have less responsibility to bear, whereas the patients in IEHT can meet obligations to family and dependents in their habitual domestic surroundings. In terms of relations to caregivers, both the interpersonal (e.g., relationship, continuity of care) and the professional therapeutic (e.g., shared decision making) levels are described as essential for the quality of collaboration in both types of treatment, for patient satisfaction and ultimately for the treatment success. As a result, the choice of the form of psychiatric treatment according to the individual needs of the patients seems to be one key driver that can in turn increase patient satisfaction, which is similar to the results of (22). A second decisive factor for patient satisfaction in IT is ensuring a clean and hygienic environment as a lack of it is one of the reasons to drop out of treatment in our sample.

Besides these comparative results, the present study corresponds in most aspects to the qualitative research results of the study group of von Peter and colleagues (21) process evaluation: in both studies, the patients perceived IEHT to be normalizing, dealing with problems close to real life, but having the risk to harm private life in different ways. The participants of both studies highly valued continuity of care as leading to more trustful relationships between staff, patients, and family. Also, need-adapted forms of treatments to choose ones' own treatment setting, i.e., shared decision making and active participation, increased patient satisfaction in both studies. Similar to the German published study of the research group of von Peter and colleagues (22), our IEHT respondents perceived the IEHT as more intense and rated it better as IT (compare also 24). Parallel are the named disadvantages as, e.g., lack of contact with fellow patients in IEHT (22).

In contrast to the quantitative study by Kirchmann and colleagues (24), our findings do not allow the conclusion that IT generally creates a higher degree of satisfaction. Rather, the results indicate a need to identify those aspects that are important to the individual patient in order to find the most appropriate type of treatment. The findings from this qualitative study indicate rather—based on their individual circumstances—that the test persons in IEHT seem to report more satisfaction with this type of therapy compared with IT. All the respondents with experience in IEHT had previously undergone IT and consider this experience as valuable, too.

Our analysis also shows that patient satisfaction in IT and IEHT is not only strengthened by a desired level of participation in decision making (28), but also seems to result in early treatment termination when not sufficiently fulfilled. Particularly with regard to staff, it is obvious that the coordination and continuity of treatment also plays an important role in patient satisfaction, while communication with staff is particularly relevant in the short term (39). This is also shown by our results regarding IT. However, we cannot conclude from our results better (mental) health outcomes due to higher patient satisfaction neither in IT nor in IEHT. But we could see that the expectations of the study participants seem to be associated with positive treatment results, which is not commonly found in recent systematic reviews (27).

The results in this qualitative study correspond almost completely to the systematic review of Batbaatar and colleagues (27) where particularly the quality of the interpersonal skills and competence of the healthcare providers and the physical environment of the facility, accessibility, and continuity of care were essential determinants with the highest influence on patient satisfaction. Nevertheless, as mentioned above, a clean and hygienic environment seems to be critical for our respondents as a lack of it is one of the reasons to drop out of treatment.

Beyond that, the participants in our study wished for peer support in their therapy, which corresponds to the study protocol of Reinke and colleagues (23) comparing home treatment with and without peer support.

### 5.1. Implications for practice

As hospitals and their wards are highly complex organizations, we cannot generalize the results of our study to other German or international hospitals. However, even small studies such as this offer important insights into potential promising practices for the delivery of patient-centered, respectful psychiatric treatment. In addition, due to the similar results of our research to those of the study group of von Peter and colleagues (21, 22) and the systematic reviews on patient satisfaction by Batbaatar and colleagues (26, 27), there seem to exist some generic issues from which German hospitals and caregivers offering IT as well as IEHT may benefit from in order to identify and better utilize existing potentials and strengths. Those are (1) continuity of care resulting in better interpersonal care and improved relationships between patient, caregiver, and family, (2) taking the needs of the patients into consideration to choose their own form of treatment, and (3) shared decision making and active participation between the patient and the caregiver, also in the process of treatment. Considering these generic issues seems to be advisable as a high level of satisfaction results in the decisions of the patients to choose a health service and in an intention to return to a particular hospital (26). However, fulfilling point (1) is currently a very critical issue as staff shortage is one of the biggest problems for all healthcare providers in Germany.

### 5.2. Strengths and limitations of the study

Certain elements of this study limit the generalization of findings. One potential factor influencing the sample is that the test persons were recruited who might have a higher satisfaction with IEHT and IT. This might bias the heterogeneity of the sample. The same applies to the fact that only women were interviewed about treatment experience. Since recruitment proved to be difficult in view of the sensitive topic explored in the study, all the test persons were included in the sample who met the inclusion criteria and showed an interest in sharing their personal experience with treatment. In line with the theoretical sampling method (35), this dominance of the female gender was therefore transferred to the sample of respondents with IEHT experience to ensure better comparability.

The qualitative, hypothesis-generating, and explorative character of the study calls for due caution in interpreting the results. But this weakness is also a strength since the qualitative approach provides an in-depth and comprehensive view on the experiences of the patients and also encourages openness for unexpected results. Moreover, this is one of the first comprehensive and profound qualitative investigations in Germany to explore and compare patient satisfaction with IT and IEHT. Nevertheless, our study suffers probably from a retrospective and social desirability bias of respondents.

Another point of criticism is the sample size of *N* = 9, which is even small also for a qualitative study. However, it proved impossible to recruit more test persons for the study, because at the time of data collection, the investigated facility had only a relatively small number of patients in IEHT.

A further weakness of our research is that we could not recruit patients with IEHT alone, i.e., without prior experience of IT. This would have added possibly more and interesting information as, e.g., some unknown “basics” are missing when patients have not previously undergone IT.

Nevertheless, the strengths of our study are that data were analyzed by multiple researchers, ensuring investigator triangulation. Moreover, two researchers had a neutral view on the data as they were not involved in the field of IT and IEHT, which is also a strength of this study. Finally, we used an interview technique that was problem-centered, which is a theory-based and long-established scientific interview method.

### 5.3. Implications for future research

Future research should compare qualitative or mixed-method studies with bigger sample sizes and a higher gender heterogeneity ideally patients in three groups of treatment: IT, IEHT with prior experience of IT, and IEHT without prior experience of IT. However, it seems to be questionable if the test subjects from various psychiatric hospitals and regions in Germany should be summarized in one data set in future studies, as hospital/wards are highly complex organizations and not directly comparable.

Beyond that, a valuable future research, particularly for healthcare practice, would be to accurately identify the different needs of the patients in IT and IEHT in larger samples in order to identify different types of patients. Resulting needs profiles and questionnaires for measuring them (40) in practice might help to better meet the needs of the patients and thus enhance patient satisfaction.

## Data Availability

The data sets presented in this article are not readily available because the authors did not include sensitive transcripts of the qualitative interviews, for the reason that the authors must preserve the anonymity of the study participants according to the German and European Data Protection Law. Requests to access the data sets should be directed to: neleadam@gmx.de.

## Footnotes

^‡^There exist rooms for the IEHT in the investigated hospital, because they offer every Friday morning a shared breakfast as an opportunity for exchange between IEHT patients.
