# Supplementary material for: Patient satisfaction in inpatient psychiatric treatment compared with inpatient equivalent home treatment in Germany: an in-depth qualitative study
Source: Front Health Serv. 2023 Jun 29;3:1195614. doi: 10.3389/frhs.2023.1195614 (PMC10344693; doi:10.3389/frhs.2023.1195614)
Supplement: Supplementary file 1 [file Table1.docx]

## Complete interview guideline

**Words of welcome for the test person and introduction to the interview**- First of all I would like to say thank you for taking the time to support me in my research project with this interview.

- My name is Nele Adam, I am a researcher at Witten/Herdecke University. Our objective in this research is to learn something about your personal experience with psychiatric treatment and about aspects or elements of this treatment which you find satisfactory and/or dissatisfactory.

- I would be pleased to hear as much as possible about your latest experience with IT or IEHT so that I can include a maximum of detailed information in my research.

- To make sure that nothing essential is overlooked, I am going to follow this interview guide. I may need to take notes during the interview, which you may of course inspect any time.

- We will now go through the declaration of consent together. Feel free to ask if something is unclear. → Detailed information on anonymity, my confidentiality statement, inclusion in the study, data protection etc.

- The interview is going to take about one hour. We can take a break whenever you wish.

- Now I need some personal information from you; afterwards I am going to turn on the recording device and start with my questions. Any questions so far?

**Interview guideline**

Test subject’s ID (first letter of place of birth + first letter of mother’s first name + last letter of father’s first name + number of test subject’s month of birth):

Time of admission:

Time of discharge:

Diagnosis:

| **Key question**  **(invitation to speak)** | **Possible follow-up questions –  only if not addressed by respondent** | **Questions to remain on topic or steer the conversation** |
| --- | --- | --- |
| 1) What was your therapy experience like? |  |  |
| 2) What did you expect from therapy? | What did you expect from caregivers (physicians, therapists)?  Which questions, wishes and needs did you have? |  |
| 3) How did you feel on the ward? / How did you feel at home?  How did you experience caregivers, fellow patients and family?  What was your experience with meals?  Which colors/smells do you remember?  Did cleanliness and hygiene contribute to your well-being? | - Atmosphere - Proximity/distance |  |
| 4) What about the treatment made you feel good? | Tell me more about these situations or methods/techniques (you learned from your therapist) which do or did you good.  What exactly did you feel?  Which positive effects did you register? | General verbal continuation: Follow-up questions   - “I don’t understand/ cannot imagine what this was like, can you give more details?” - What do you mean by that? - How did this feel? - Could you describe this more precisely? - What exactly was the situation? - Which persons were involved? |
| 5) What did you feel in the situations that were good for you? | - Did you feel safe, oriented? - Did you feel proud? - Did you receive praise? - Did you feel appreciated? - Treated with respect? - Were you pleased? - Were your mental and physical boundaries respected? - Did you have the impression you were taken seriously? - Did you decide/have a say in how to arrange your treatment? - Did you feel well supported/taken care of/advised/understood? |  |
| 6)Which aspects of therapy were not so good for you? | Please describe in detail those situations or methods/techniques (learned from the therapist) which are or were not good for you. | General verbal continuation: follow-up questions   - “I don’t understand/ cannot imagine what this was like, can you give more details?” - What do you mean by that? - How did this feel? - Could you describe this more precisely? - What exactly was the situation? - Which persons were involved? |
| 7) How did you feel in situations that were not so good for you? | - Did you feel insecure, disoriented? - Did you feel ashamed? - Were you threatened or sanctioned? - Did you feel hurt? - Humiliated? - Disrespectfully treated? - Were you frightened? - Did you feel unappreciated? - Were your mental and/or physical boundaries exceeded? - I did not feel taken seriously - I had to give way - Did your therapist reproach you? - Did you feel abandoned? |  |
| Is it possible that unfavorable circumstances (setting, environment, caregivers) had a negative effect? | - Possibly your mental state - Relation to the therapist - Therapy terminated? - Or something else? |  |
| Did they use physical restraints on you? | How did you experience that?  How did you deal with that (at the time / today)?  Do you believe the use of restraints made sense in that situation? |  |
| Looking back, what was essential to the treatment outcome? | - Did you feel recovered as a result of treatment? - Did you feel perceived and taken seriously as a person in the treatment context? - Did you perceive an improvement of your state of health as a result of treatment? - Would you say you have gained valuable /positive/enjoyable experiences during treatment? |  |
| Would you opt for this kind of treatment again?  Would you recommend the treatment to your best friend? | - Why exactly would(n’t) you make the same decision a second time? - Why would(n’t) you make that recommendation? | General verbal continuation: follow-up questions   - “I don’t understand/ cannot imagine what this was like, can you give more details?” - What do you mean by that? - How did this feel? - Could you describe this more precisely? |
| What would you improve if you had the chance?  In case of consistently positive experiences: What has improved?  What is your notion of ideal treatment? |  | General verbal continuation: follow-up questions   - I don’t understand/ cannot image what this was like, can you give more details? - What do you mean by that? - Could you describe this more precisely? - What exactly is your idea of the therapy situation? |
| Is there anything you would like to add or mention what has not been said or addressed so far? |  |  |

- Thank you very much for your effort and for your support to my research project.
